# Continuous extracorporeal treatments in a dialysis patient with COVID-19

**DOI:** 10.1007/s13730-020-00538-x

**Published:** 2020-10-04

**Authors:** Yoshihito Nihei, Hajime Nagasawa, Yusuke Fukao, Masao Kihara, Seiji Ueda, Tomohito Gohda, Yusuke Suzuki

**Affiliations:** Department of Nephrology, Faculty of Medicine, Juntendo University, 2-1-1 Hongo, Bunkyo-ku, Tokyo, 113-8421 Japan

**Keywords:** COVID-19, Plasma exchange, Continuous renal replacement therapy, Cytokine storm

## Abstract

The coronavirus disease 2019 (COVID-19) pandemic is now a major global health threat. More than half a year have passed since the first discovery of severe acute respiratory syndrome coronavirus-2 (SARS-CoV2), no effective treatment has been established especially in intensive care unit. Inflammatory cytokine storm caused by SARS-CoV-2 infection has been reported to play a central role in COVID-19; therefore, treatments for suppressing cytokines, including extracorporeal treatments, are considered to be beneficial. However, until today the efficacy of removing cytokines by extracorporeal treatments in patients with COVID-19 is unclear. Herein, we report our experience with a 66-year-old male patient undergoing maintenance peritoneal dialysis who became critically ill with COVID-19 and underwent several extracorporeal treatment approaches including plasma exchange, direct hemoperfusion using a polymyxin B-immobilized fiber column and continuous hemodiafiltration. Though the patient developed acute respiratory distress syndrome (ARDS) repeatedly and subacute cerebral infarction and finally died for respiratory failure on day 30 after admission, these attempts appeared to dampen the cytokine storm based on the observed decline in serum IL-6 levels and were effective against ARDS and secondary haemophagocytic lymphohistiocytosis. This case suggests the significance of timely initiation of extracorporeal treatment approaches in critically ill patients with COVID-19.

## Introduction

Since the diagnosis of the first patient in December 2019 in Wuhan, China, coronavirus disease 2019 (COVID-19), which is caused by severe acute respiratory syndrome coronavirus-2 (SARS-CoV-2) infection, has evolved into a pandemic [1]. As of 31 July 2020, over 17.5 million people have been infected with SARS-CoV-2 and more than 660, 000 individuals have died. SARS-CoV-2 infection leads to various syndromes including acute respiratory distress syndrome (ARDS), secondary haemophagocytic lymphohistiocytosis (sHLH) and venous and arterial thromboembolic disease, especially in patients in the intensive care unit. The cytokine storm caused by SARS-CoV-2 infection, primarily characterised by elevated plasma concentrations of interleukin 6 (IL-6), plays a central role in COVID-19 [2]; therefore, its suppression is considered a key treatment approach in patients with COVID-19.

Extracorporeal treatment approaches including plasma exchange (PE), direct hemoperfusion using a polymyxin B-immobilised fibre column (PMX-DHP) and continuous hemodiafiltration (CHDF) have been used to remove inflammatory cytokines [3, 4]. Especially, CHDF is reported to continually suppress inflammatory cytokines and has been used in critically ill patients, including those with septic shock, ARDS and infections with viruses such as severe acute respiratory syndrome coronavirus and Middle East respiratory syndrome coronavirus [5]. Recently, Yang et al. demonstrated that CHDF might improve all-cause mortality in patients with COVID-19 undergoing mechanical ventilation. However, the efficacy of extracorporeal treatment approaches in patients with COVID-19 is unclear. Herein, we present a patient on peritoneal dialysis (PD) who became critically ill with COVID-19 and was treated with continuous extracorporeal treatments including PE, PMX-DHP and CHDF. We also discuss the efficacy of these treatments in patients with COVID-19.

## Case report

**Fig. 1:**
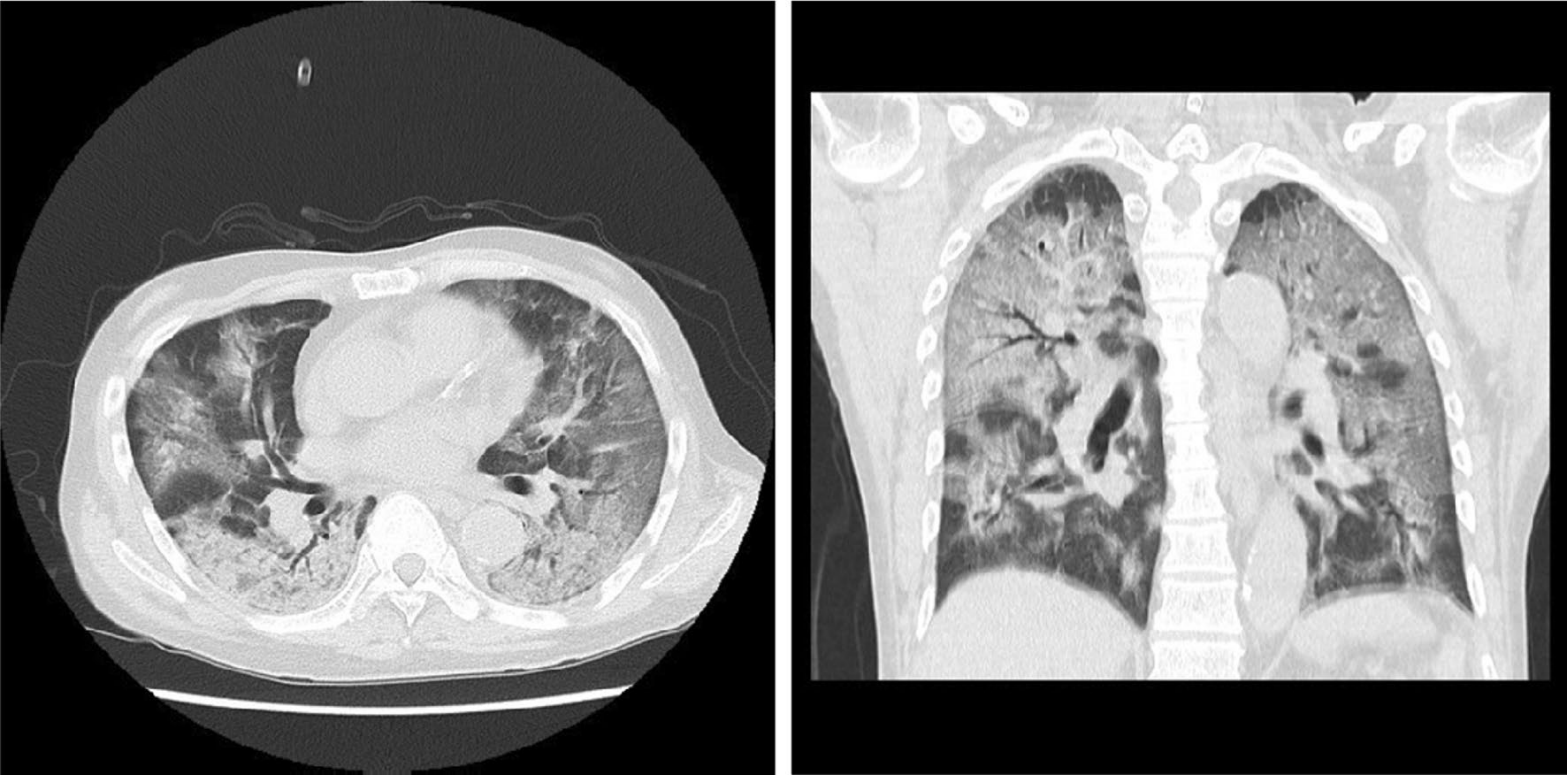
Chest computed tomography scan on admission

A 66-year-old male patient on PD due to end-stage renal failure (ESRD) with IgA nephropathy developed a fever of 38 ℃ with cough and fatigue three days before admission and was admitted to Juntendo University Hospital with cough, severe respiratory distress and a body temperature of 39 ℃. Laboratory tests revealed lymphocytopenia and elevated C-reactive protein, ferritin and D-dimer levels on admission (Table 1). High-resolution computed tomography (CT) of the chest showed massive ground-glass opacities (GGO) in bilateral lungs (Fig. 1), leading to the suspicion of COVID-19 with ARDS. The nasopharyngeal swab sample was positive for SARS-CoV-2 by quantitative polymerase chain reaction (qPCR), and the patient was diagnosed with COVID-19. He required intubation and mechanical ventilation on day one because of worsening hypoxemia and admitted to the intensive care unit for further evaluation and management.

**Table 1:** Biochemical and biomarker test results on admission and before onset

	On admission	Before onset
White blood cell count (× 10^3^ cells per μL)	10.1	6.98
Lymphocyte count (× 10^3^ cells per μL)	0.67	N.D
Hemoglobin (g/dL)	13	12.6
Platelet count (× 10^3^ cells per μL)	318	283
Blood urea nitrogen (mg/ dL)	106	61
Creatinine (mg/dL)	21.82	15.4
Total Bilirubin (mg/dL)	0.2	N.D
Aspartate aminotransferase (U/L)	48	17
Alanine aminotransferase (U/L)	20	11
Lactate dehydrogenase (U/L)	946	N.D
Creatine kinase (U/L)	124	155
d-dimer (μg/mL)	10.8	N.D
FDP (μg/mL)	9.1	N.D
Prothrombin time (s)	20.2	N.D
Ferritin (ng/mL)	6328	N.D
CRP (mg/dL)	12.99	0.3
Procalcitonin (ng/mL)	2.7	N.D
IL-6 (pg/mL)	128	N.D
KL-6 (U/mL)	861	N.D
IgG (mg/dL)	527	N.D
BNP (pg/ml)	131.7	99.8

*FDP* fibrinogen degradation products; *CRP* C-reactive protein; *IgG* immunoglobulin G; *IL-6* interleukin 6; *N.D.* not determined; *BNP* brain natriuretic peptide

**Fig. 2:**
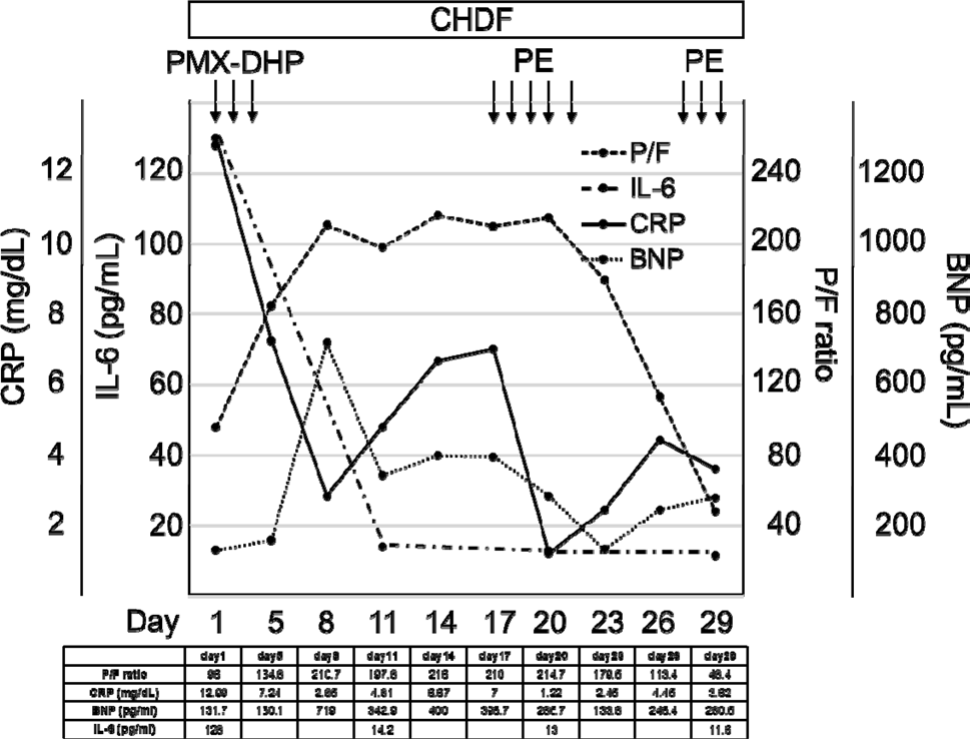
Extracorporeal treatments for acute respiratory distress syndrome caused by coronavirus disease 2019**.** *CHDF* continuous hemodiafiltration; *PMX-DHP* polymyxin B-immobilised fibre column; *PE* plasma exchange; *P/F* PaO_2_/FiO_2_ ratio; *CRP* C-reactive protein; *IL-6* interleukin 6; *BNP* brain natriuretic peptide

He was started on hydroxychloroquine (400 mg/day for 14 days) to suppress viral replication and methylprednisolone (1 mg/kg) for the treatment of ARDS. Given that the patient was on PD, CHDF with a CH1.8-W (polymethyl methacrylate membrane) was initiated on day one and maintained during the entire clinical course. As we could not measure his body weight, we decided the dry weight according to the levels of blood pressure and BNP (Fig. 2). As anticoagulants, we used Nafamostat instead of heparin because it was reported to have the potential to inhibit the inflammatory reactions caused by SARS-CoV-2 [6]. While early CHDF initiation has been reported to be effective in removing inflammatory cytokines in patients with ARDS, its efficacy remains controversial [7]. Therefore, for a stronger suppression of the cytokine storm, PMX-DHP (2 h per day for 3 days) was started on day three. As shown Fig. 2, the initiation of PMX-DHP led to a reduction in serum IL-6 levels and improved the PaO_2_/FiO_2_ ratio from 95 on day one to 200 on day eight. Since the serum IgG levels were low, the patient received IVIg (5 g/day) for three consecutive days from day five to day seven.

**Fig. 3:**
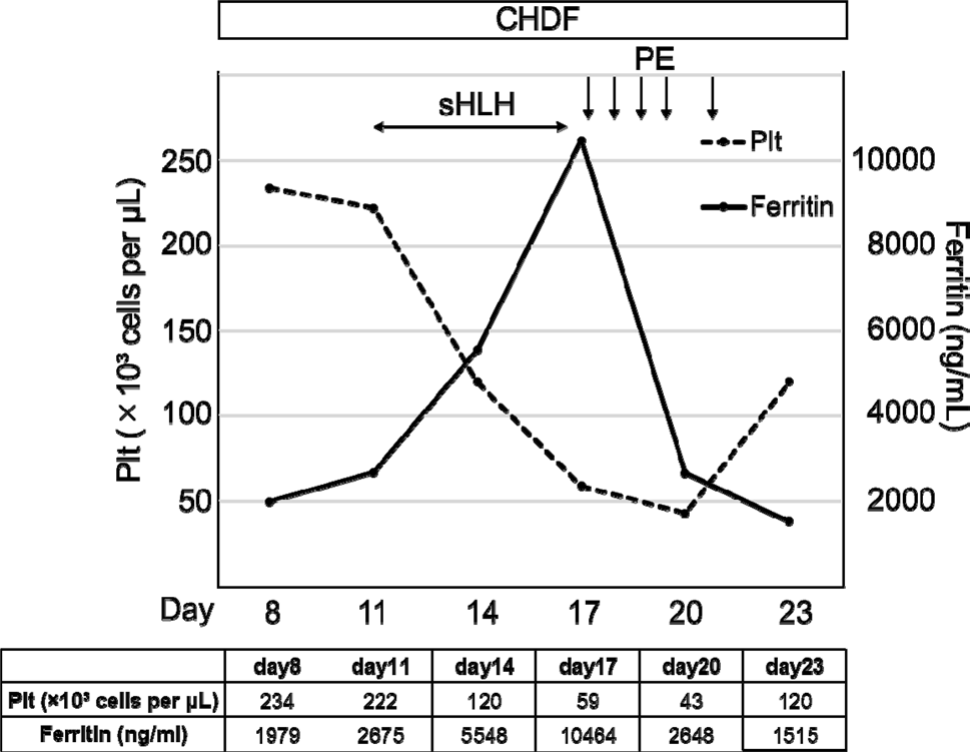
Extracorporeal treatments for sHLH caused by COVID-19**.** *CHDF* continuous haemodiafiltration; *PE* plasma exchange; *Plt* platelets; *sHLH* secondary haemophagocytic lymphohistiocytosis

Starting on day 11, he developed sHLH based on the presence of fever, thrombocytopenia and elevated levels of ferritin, lactate dehydrogenase. His symptoms worsened in the absence of evidence for bacterial or fungal infection; therefore, he was considered to have developed sHLH due to an uncontrolled cytokine storm caused by COVID-19, although his serum IL-6 levels were not determined after the development of sHLH. As PE was reported to be effective in sHLH, it was performed with replacement by 2880 ml of fresh frozen plasma for three consecutive days from day 17 to day 19, with two subsequent PEs performed on day 20 and 22. Thrombocytopenia and hyperferritinemia rapidly improved after the initiation of PE (Fig. 3), suggesting its potentially protective effect against sHLH caused by COVID-19.

**Fig. 4:**
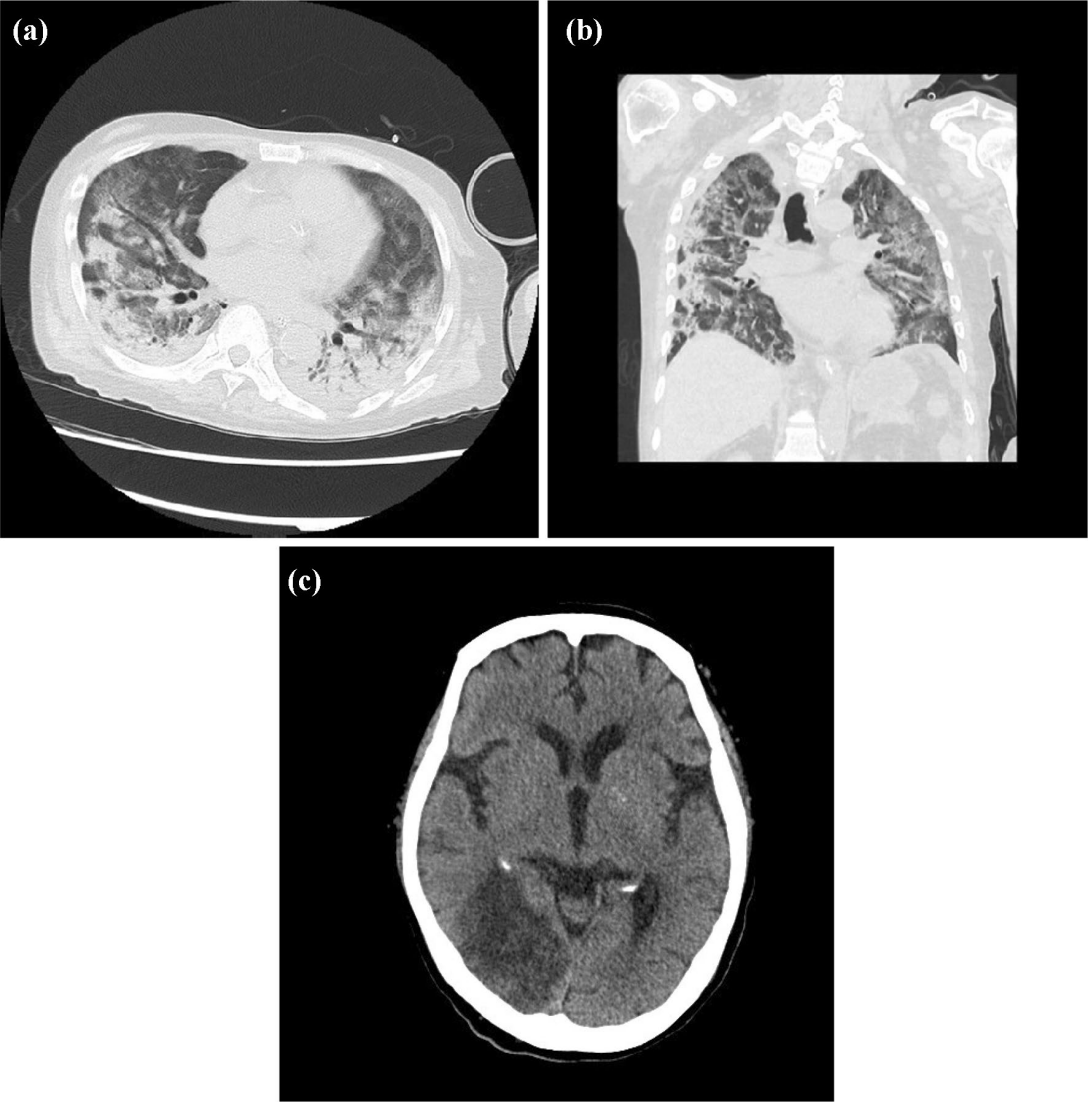
Computed tomography scans on day 26. **a, b** Chest computed tomography scan. **c** Cranial computed tomography scan

The patient was started on the viral RNA polymerase inhibitor favipiravir (1800 mg on day one and 800 mg/day for the next 13 days) on day 14 after its approval in Japan; however, the sputum sample remained positive for SARS-CoV-2 by qPCR. Chest X-ray obtained on day 26 showed diffuse bilateral coalescent opacities, and the PaO_2_/FiO_2_ ratio drastically decreased from 180 to 100 within 48 h and high-resolution CT scan showed worsening GGO (Fig. 4), suggesting that the patient developed ARDS a second time. Although the patient did not show any obvious symptoms suggestive of stroke because he was under sedation, cranial CT scan revealed subacute infarction in the right occipital/temporal lobe (Fig. 4) despite continuous administration of anticoagulants after the patient’s admission to the intensive care unit. PE and methylprednisolone were reinitiated to suppress the cytokine storm and to treat ARDS, respectively (Fig. 2). However, the patient developed respiratory failure and died on day 30 after admission. His blood pressure level was in the normal range until the day he died, suggesting the absence of shock during the clinical course.

## Discussion

Since its first identification, SARS-CoV-2 infection has spread rapidly across the globe. Although there are no confirmed treatment approaches for COVID-19, a combined approach by controlling viral replication and suppressing the cytokine storm is considered as an important therapeutic strategy for COVID-19. We herein present a patient on PD who became critically ill due to COVID-19 and was treated with several extracorporeal treatments including PE, PMX-DHP and CHDF to suppress the cytokine storm. Although the dysregulation of inflammatory cytokines could not be completely suppressed, these extracorporeal treatments were effective against ARDS and sHLH, suggesting the significance of extracorporeal treatments in suppressing cytokine storm in COVID-19.

**Fig. 5:**
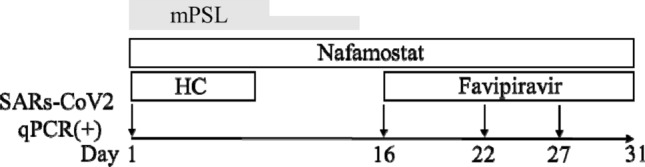
Positivity for SARS-CoV-2 throughout the course of treatment by quantitative polymerase chain reaction. *HC* hydroxychloroquine; *mPSL* methylprednisolone 1 mg/kg from day 1 to day 8 and 0.5 mg/kg from day 9 to day 14; *SARS-CoV-2* severe acute respiratory syndrome coronavirus-2

Studies on patients with COVID-19 in China showed that a high viral load correlated with worse symptoms and that SARS-CoV-2 was detectable until death in non-survivors [8], highlighting the importance of controlling viral replication for the treatment of COVID-19. Regarding antiviral therapy, we failed to effectively reduce viral replication in the present patient. Antiviral drugs favipiravir could not be initiated at an early stage of treatment as they had not been approved in Japan at the time. Favipiravir was initiated on day 14 after admission, immediately after its approval for use in Japan. However, the persistent positivity of the sputum samples for SARS-CoV-2 throughout the course of treatment (Fig. 5) indicated that the viral replication could not be completely suppressed by hydroxychloroquine and favipiravir. CHDF has been reported to adsorb several types of drugs including favipiravir [9] and might have reduced the antiviral activity of favipiravir. Therefore, appropriate drug doses, administration methods and drug monitoring should be considered more carefully in patients undergoing CHDF. Additionally, corticosteroids used for ARDS in the present patient might also have led to delayed viral clearance.

COVID-19 has been reported to arise from an inflammatory cytokine storm due to SARS-CoV-2 infection; therefore, suppression of the cytokine storm is a key approach for the treatment of patients with COVID-19. Extracorporeal treatment approaches have been used to remove inflammatory cytokines in patients with septic shock and ARDS, whereas several studies have reported the efficacy of PE, PMX-DHP and CHDF in patients with COVID-19 [10]. In the present case, three extracorporeal treatment approaches were utilised. As the patient was on PD due to ESRD, CHDF was started on day one and continued during the entire clinical course. The efficacy of CHDF is controversial, although it has been reported to be beneficial in patients with ARDS by reducing the levels of inflammatory cytokines [7]. Therefore, we performed PMX-DHP, which has also been demonstrated to adsorb various cytokines. CHDF and PMX-DHP increased the PaO_2_/FiO_2_ ratio (Fig. 2) in the present patient, indicating their efficacy in ARDS caused by COVID-19. Several studies have revealed that IL-6 plays a key role in cytokine storm and that serum IL-6 levels correlate with the severity of COVID-19. In the present case, serum IL-6 levels decreased after the treatment initiation and remained low during the clinical course (Fig. 2), suggesting the possibility that uninterrupted CHDF treatment might have at least suppressed the elevation of serum IL-6 levels.

sHLH is commonly reported in patients with severe COVID-19 [11]. Consistently, the present patient developed sHLH on day 11 after admission. We did not find evidence of other diseases that could cause inflammation, including bacterial, viral and fungal infections, suggesting that the cytokine storm caused by COVID-19 was not completely controlled. Effective treatment of sHLH requires aggressive immunosuppression with agents such as corticosteroids and cyclosporin to control the hyperinflammatory state. These immunosuppressive treatments were not used in the present patient as they could further delay viral clearance. Given that the cytokine storm causes sHLH, the PE initiated on day 17 to remove cytokines rapidly improved thrombocytopenia and hyperferritinemia (Fig. 3), suggesting that PE was protective against sHLH. Taken together, the extracorporeal treatment approaches employed in the present patient were effective in the treatment of ARDS and sHLH by suppressing the cytokine storm caused by COVID-19.

In addition to the failure in the control of viral replication, abnormal immune response due to ESRD might also have contributed to the incomplete suppression of the cytokine storm by the extracorporeal treatment approaches utilised in the present case. Patients with ESRD exhibit dysfunction in a variety of immune cells [12]. CD8^+^ T cells play an important role in a variety of viral infections, including SAR2-CoV-2 infection. Loss of renal function has been reported to decrease both the total number and the composition of circulating CD8^+^ T cells, indicating that patients with ESRD have reduced ability to eliminate virus. Therefore, it remains possible that the number and function of the CD8^+^ T cells might have been insufficient to clear SARS-CoV-2 in the present patient.

**Fig. 6:**
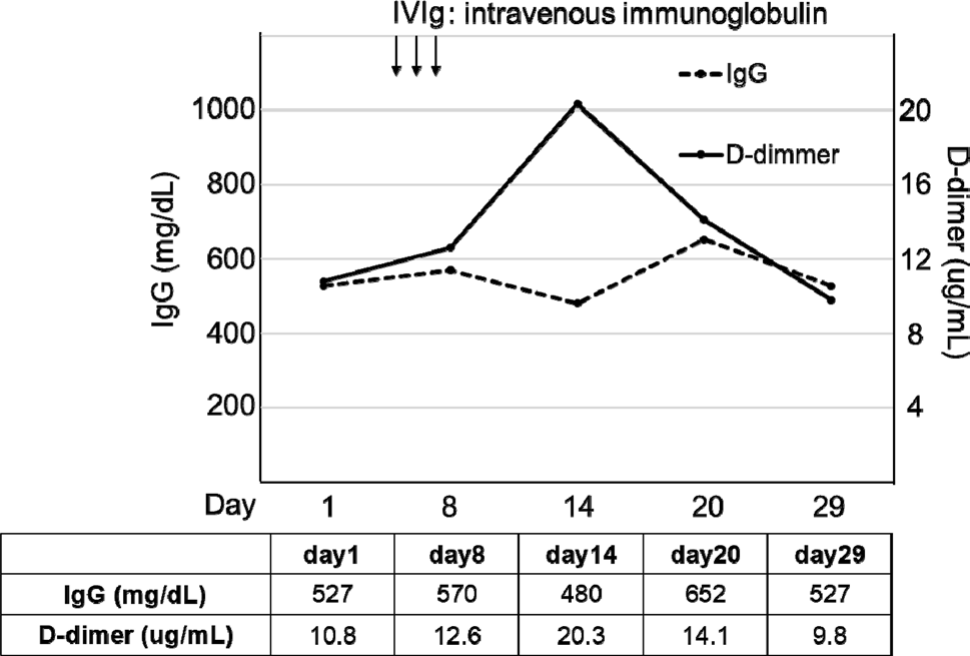
Continuous hypogammaglobulinemia and hypercoagulability. *IVIg* intravenous immunoglobulin

B cells are another immune cell type that plays an important role in protection from viral infections by generating non-specific as well as virus-specific antibodies. Patients with common variable immunodeficiencies and defective antibody production have been reported to exhibit severe symptoms of COVID-19 [13]. The present patient showed a marked decrease in circulating CD19^+^ B cell numbers (at day 16, 0.5% per CD45 + cell; 10% or more is normal), which did not recover during the clinical course (at day 28, 1.8% per CD45 + cell). Moreover, his serum IgG levels remained low despite the administration of intravenous immunoglobulin (Fig. 6). Another report showed that early intravenous immunoglobulin administration was protective in critically ill patients with COVID-19 [14], suggesting that a low gamma globulin level in early-stage COVID-19 might correlate with worse outcomes. Given these reports, an abnormal humoral immunity might have exacerbated the clinical condition of the present patient. However, it remains unclear why serum immunoglobulin levels were already low at the time of admission. One potential explanation is that SARS-CoV-2 might have a direct effect on B cells. As an abnormal humoral immune response might have exacerbated the cytokine storm in the present patient, earlier initiation of extracorporeal treatment approaches might have been more effective suppressing the cytokine storm. Further studies are necessary to clarify this possibility.

The CT scan conducted on day 28 revealed subacute cerebral infarction. SARS-CoV-2 infection can cause hypercoagulability [15], and the serum D-dimer levels remained high during the clinical course despite the continuous administration of the anticoagulant nafamostat in the present patient (Fig. 6). The uncontrolled cytokine storm and viral replication might have exacerbated the hypercoagulability, which might be an underlying cause of the fatal outcome in the present patient. In patients with high D-dimer levels despite anticoagulant therapy, attention should be paid to thrombosis.

In summary, we herein presented a patient on PD who became critically ill with COVID-19 and was treated with several extracorporeal treatment approaches including PE, PMX-DHP and CHDF. These extracorporeal treatments were somewhat effective in suppressing the cytokine storm; however, the patient eventually died of an uncontrolled immune response and hypercoagulability. The cytokine storm might not have been suppressed at all without extracorporeal treatments, highlighting their significance in suppressing the cytokine storm during COVID-19. In addition to treatments suppressing abnormal immune response caused by SARS-CoV-2 infection, timely initiation of extracorporeal treatment approaches may be beneficial in critical ill patients with COVID-19.
